# Using the Community Readiness Model to Examine the Built and Social Environment: A Case Study of the High Point Neighborhood, Seattle, Washington, 2000–2010

**DOI:** 10.5888/pcd11.140235

**Published:** 2014-11-06

**Authors:** Joyce Buckner-Brown, Denise Tung Sharify, Bonita Blake, Tom Phillips, Kathleen Whitten

**Affiliations:** Author Affiliations: Denise Tung Sharify, Neighborhood House Community Health Program, Seattle, Washington; Bonita Blake, High Point Community Council, Seattle, Washington; Tom Phillips, Developer, High Point Community, Seattle Washington; Kathleen Whitten, ICF International, Atlanta, Georgia.

## Abstract

**Background:**

Residents of many cities lack affordable, quality housing. Economically disadvantaged neighborhoods often have high rates of poverty and crime, few institutions that enhance the quality of its residents’ lives, and unsafe environments for walking and other physical activity. Deteriorating housing contributes to asthma-related illness. We describe the redevelopment of High Point, a West Seattle neighborhood, to improve its built environment, increase neighborhood physical activity, and reduce indoor asthma triggers.

**Community Context:**

High Point is one of Seattle’s most demographically diverse neighborhoods. Prior to redevelopment, it had a distressed infrastructure, rising crime rates, and indoor environments that increased asthma-related illness in children and adolescents. High Point residents and partners developed and implemented a comprehensive redevelopment plan to create a sustainable built environment to increase outdoor physical activity and improve indoor environments.

**Methods:**

We conducted a retrospective analysis of the High Point redevelopment, organized by the different stages of change in the Community Readiness Model. We also examined the multisector partnerships among government and community groups that contributed to the success of the High Point project.

**Outcome:**

Overall quality of life for residents improved as a result of neighborhood redevelopment. Physical activity increased, residents reported fewer days of poor physical or mental health, and social connectedness between neighbors grew. Asthma-friendly homes significantly decreased asthma-related illness among children and adolescents.

**Interpretation:**

Providing affordable, quality housing to low-income families improved individual and neighborhood quality of life. Efforts to create social change and improve the health outcomes for entire populations are more effective when multiple organizations work together to improve neighborhood health.

## Background

The relationship between health disparities and the built environment has been the focus of recent research examining new approaches to the ongoing challenge of providing affordable, quality housing in low-income neighborhoods (1–8). Housing is one of the 11 social determinants of health (9) because the built environment has measurable effects on both physical and mental health and can encourage physical activity, improve access to healthful foods, and reduce crime through neighborhood design and regulations (1–8).

According to the US Surgeon General, healthy homes are those “sited, designed, built, renovated, and maintained in ways that support the health of residents. Specific features that constitute healthy housing include structural and safety aspects of the home (ie, how the home is designed, constructed, and maintained; its physical characteristics; and the presence or absence of safety devices), quality of indoor air and water, and the presence or absence of chemicals” (8). In contrast, poor housing can expose people to dangerous physical conditions. Environmental health science studies include not only the effects of pollutants, but also other factors that affect population health, such as housing quality and other indicators of social distress (2). Differences in neighborhoods and housing quality among people of different socioeconomic status add to the disproportionate burden of illness and injury among minority racial/ethnic populations and low-income communities (2). For example, asthma has become a major public health issue in urban areas because of its increasing prevalence and disproportionate effect on children and adolescents in low-income and minority urban communities. Dilapidated housing is associated with asthma triggers such as mold, moisture, dust mites, and rodents, and with mental health stressors such as violence and social isolation (2). This article describes how a Seattle, Washington, community implemented a comprehensive redevelopment plan that created a sustainable built environment with improved indoor environmental quality.

## Community Context

High Point, in the Delridge district of West Seattle, is one of the city’s most demographically diverse neighborhoods and has a substantial immigrant population from Southeast Asia and East Africa (10). Before community redevelopment, High Point was a culturally diverse community with many racial/ethnic groups: 36% of residents were African or African-American, 29% were Asian/Pacific Islander, 18% were non-Hispanic white, and 17% were other races/ethnicities. Most household heads (61%) were born outside the United States. The neighborhood’s cultural diversity was also reflected in language: only 37% of residents spoke English as their preferred language; 26% spoke Vietnamese, 12% spoke Cambodian, 8% spoke Somali, and the remaining 17% spoke 1 of 10 other languages (10). The High Point community was developed in 1942 to provide temporary government housing to defense workers during World War II (10). The Seattle Housing Authority assumed administrative oversight in 1953 and converted High Point into public housing (10). By the late 1960s, High Point had a distressed infrastructure and rising crime rates. The indoor environments of its residences placed children at substantial risk of exposure to asthma triggers. Many families with children experienced repeated visits to emergency departments for asthma-related illness.

The High Point redevelopment initiative was undertaken with the overall objective of improving neighborhood quality of life with community involvement. The aim of community involvement was to promote collaboration among all stakeholders in the redevelopment effort, including residents, federal and local government, and private funders. We describe the collaboration that made the redevelopment possible, the built environment that increased the walkability of the neighborhood, and pediatric asthma outcomes.

## Methods

From the start, the Seattle Housing Authority engaged High Point residents and community organizations in the redevelopment planning process. Through meetings and collaboratively designed workshops, residents and planners focused on making High Point a home for people of all ages and cultures. Residents were involved in every aspect of planning, including problem identification, strategy development and implementation, and program evaluation. We conducted a retrospective analysis of the project and used the 9-stage Community Readiness Model developed by the Tri-Ethnic Center for Prevention Research at Colorado State University (www.triethniccenter.colostate.edu) to describe the mobilization of High Point’s collaborative efforts (11,12) (Table 1).

**Table 1:** Stages of Community Readiness to Address Issues of Housing and Environment, High Point Community Redevelopment, Seattle, Washington, 2000–2010

Stage	Status of Awareness of Issues	Goal	Status of Housing and Environment
No awareness	Issue is not generally recognized by the community or leaders as a problem (or it may not be an issue).	Raise awareness of the issue.	In 2000, High Point was more than 60 years old and in varying stages of deterioration; pediatric emergency department visits for asthma were common, and gang-related activity had escalated.
Denial or resistance	At least some community members recognize that housing and environment are a concern, but there is little recognition that they may be local issues.	Raise awareness that the problem or issue exists in this community.	Not observed
Vague awareness	Most acknowledge a local concern, but there is no immediate motivation to address concerns.	Raise awareness that the community can do something.	In 2001, 537 High Point residents completed a residential needs assessment.
Preplanning	Residents recognize that something must be done, and there may even be a group addressing the issue. However, efforts are not focused or detailed.	Raise awareness with concrete ideas to combat condition.	• In 2000 Seattle Housing Authority was awarded $35 million in redevelopment funding by US Department of Housing and Urban Development HOPE VI program. • New community partnerships were developed (eg, Partnership for High Point’s Future was formed to advise the Seattle Housing Authority on the redevelopment of High Point). • Builders purchased property at High Point; this purchase provided an additional $165 million in funds, bringing the total redevelopment project total to $200 million. • Academic consultants with expertise in social and built environments assisted the High Point coalition in structural design.
Preparation	Leaders begin planning in earnest. Community offers modest support of efforts.	Gather information with which to plan strategies.	• Partnership used community-based participatory approaches to guide its formation and operation. • Community action teams convened to assess community conditions, discuss community concerns, build leadership and social capital, and develop activities to address built and social environmental challenges. • The Seattle Housing Authority Board of Commissioners approved a replacement housing plan for residents for the High Point redevelopment.
Initiation	Enough information is available to justify redevelopment efforts. Activities are under way.	Provide community-specific information to residents.	• Phase I of the redevelopment began in 2003 and was completed in December 2007. • Phase II began in 2006 and was completed in 2010. • All housing was constructed to Built Green^b^ standards. • Principles of the New Urbanism guided the redevelopment process. This urban design concept promotes social interaction, creates walkable spaces, and supports physical safety.
Stabilization	Activities are supported by administrators or community decision makers. Staff are trained and experienced.	Stabilize efforts and programs.	• The renovation of High Point resulted in a green and sustainable community. • The neighborhood redevelopment has been recognized with prestigious awards for land use and development achievement. • The community engagement process created housing with health as its focus.
Confirmation/expansion	Efforts are in place. Community members feel comfortable using services, and they support Phase II expansions^c^. Local data are regularly obtained.	Expand and enhance services.	• In 2010, High Point had nearly 1,700 new affordable and market-rate units. • Proceeds from the land and home sales have helped fund low-income housing in the neighborhood and elsewhere. • Land has been returned to the city’s property tax rolls, where it can generate revenues to help keep the neighborhood economically self-sufficient.
High level of community ownership	Detailed and sophisticated knowledge exists about prevalence, causes, and consequences. Evaluation guides new directions for redevelopment. The High Point model is applied to other issues (ie, residents continuing vigilance, functioning as stewards of the neighborhood).	Maintain momentum and continue growth	• High Point’s redevelopment success resulted from the close cooperation between planners, residents, and other community stakeholders. • Continuous accountability and oversight are crucial and are the responsibility of all residents, to make or amend community policy recommendations as needed. • One study compared utility rates of similar properties to High Point and found High Point residents are paying 30% less. • The proportion of residents with urgent asthma-related clinical visits decreased from 62% to 21% in a 3- month period. • Walking groups have emerged and are meeting current physical activity guidelines.

a Adapted from Plested et al (12).

b Built Green is set of standards of excellence that can have a significant effect on housing, health, and the environment (http://www.epa.gov/greenbuilding/index.htm). These standards promote healthier living, greater energy savings, and a greener environment.

c Phase II expansions include, for example, for-sale homes, a community center that offered child care and employment placement services, and an assisted living facility (Elizabeth House).

### Awareness of the problem

By summer 2000, High Point was more than 60 years old and in varying stages of deterioration. The original development consisted of 1,300 units. By the 1970s, 550 units had been demolished because of poor condition or because they were located in landslide-prone areas. By 2000, 716 of the remaining units were occupied by public housing residents; the rest housed social service providers (10). Community members reported an increase in gang activity and a crack cocaine epidemic. Asthma was so commonplace it was considered normal (13). Mold developed from leaking windows that had soaked the plasterboards over the course of years. Dust mites and roaches, common asthma triggers, were prevalent (14). Residents were aware of the problems but lacked the resources to make changes.

### Preplanning

In the 1990s the Seattle Housing Authority began applying for funding from the Housing Opportunities for People Everywhere VI (HOPE VI) (10), a US Department of Housing and Urban Development (HUD) program to revitalize severely distressed public housing. In 2000 the Seattle Housing Authority was successful in obtaining $35 million for redevelopment of High Point under HOPE VI. The High Point coalition, a group of 30 to 40 High Point residents, was formed to advise the Seattle Housing Authority on the redevelopment of High Point. In addition to community members, other members of the coalition were the City of Seattle, the Seattle Health Department, Rotary Club, the High Point Community Council, Sunrise Heights Neighborhood Association, Morgan Community Association, the Delridge and Southwest District Council, High Point Neighbors, Highland Park Action Committee, West Seattle Chamber of Commerce, West Seattle Kiwanis, and the King County school district. Partners met monthly to determine project goals, design strategies, oversee implementation and evaluation methods, and review evaluation findings (10). High Point residents received stipends for participating and represented the community at meetings.

Seattle’s mayor and city council members were champions for the High Point redevelopment initiative. Community leaders in West Seattle circulated a petition and contacted civic and government officials to obtain their support for the project.

A local business partner provided tax credit syndicators. (A syndicator acquires low-income housing tax credits from affordable housing developers by investing equity in their housing developments in exchange for their tax credits.) Builders purchased property at High Point, which provided an additional $165 million to the project budget, bringing the total project budget to $200 million. Three consultants from University of Washington with expertise in social and built environments assisted the High Point coalition in structural design.

Seattle Housing Authority conducted a needs assessment in the summer of 2001. Of 609 households contacted, slightly more than a third (34%) said that people at High Point had problems with alcohol, and 41% said they thought that High Point residents had a problem with tobacco (10). Most residents (77%) said they thought that High Point was a safe place. Among minority residents who said that High Point was unsafe, the most frequently problems cited were drug dealing or drug use (80%), outsiders causing trouble (77%), gangs (74%), noise (61%), and car vandalism (60%) (10).

### Preparation

The High Point revitalization project was developed and implemented by a partnership of residents, community organizations, Seattle Housing Authority staff, public health practitioners, university faculty, and other public agencies. The coalition intentionally used community-based participatory approaches to guide its formation and operation (15). The values that guided its work included development of community capacity and equitable partnerships, linguistic and ethnic inclusivity, and community ownership. Equitable partnerships require sharing knowledge, power, and resources and providing reciprocal appreciation of what each group offers the partnership. For example, High Point’s Community Council identified asthma as a problem in the community and, in collaboration with the structural design experts, developed the residences as “Breathe Easy” units (http://www.seattlehousing.org/redevelopment/high-point/breathe-easy/).

The coalition supported the development of youth and adult community action teams. Each team consisted of 8 to 10 community members who convened to assess community conditions, discuss community concerns, build leadership and social capital, and develop activities to address changes to the built and social environment. More than 450 High Point residents and their West Seattle neighbors participated in a design survey and hands-on workshop that considered what the redeveloped High Point should look like. The Seattle Housing Authority Board of Commissioners approved the High Point redevelopment plan to serve residents whose incomes were below 30% of HUD’s area median income for King County.

### Initiation

The redevelopment of High Point occurred in 2 phases. Phase I began in 2003 and was completed in December 2007. Phase I consisted of construction of new public housing units. Phase II, infrastructure construction, began in 2006 and was completed in 2010 (Table 2). An integrated team of architects, interior designers, landscape architects, urban designers and planners, High Point residents, West Seattle community members, and Seattle Housing Authority staff worked together to create High Point’s redevelopment plan. The plan included 3 major components: quality design, a healthy environment, and an engaged community. The redevelopment plan created a safe, high-quality, and healthy residential environment with a range of housing types, each constructed to Built Green standards (http://www.epa.gov/greenbuilding/index.htm) and fully integrated with the surrounding community (13). New streets were realigned and reconnected with the West Seattle grid, and new neighborhood facilities and community services were operating at more inviting locations. The mix of housing types and resident income levels became more compatible with the greater neighborhood. Principles of New Urbanism guided the redevelopment process (16). New Urbanism is a revitalized urban design system that promotes social interaction, creates walkable spaces, and supports physical safety (eg, sidewalk-facing porches, windows facing streets to allow observation) (17,18).

**Table 2:** Redevelopment Timeline of High Point, Seattle, Washington, 2000–2010^a^

Year	Milestone
**2000**	High Point community is awarded $35 million in HOPE VI^b^ funding for redevelopment.
**2001**	Relocation counseling and assistance for more than 700 High Point households begins.
**2003**	The Seattle City Council approves High Point’s master plan. Demolition of old public housing units starts.
**2005**	Families return to new public housing units in Phase I. Construction of for-sale homes begins.
**2006**	Families continue to move into finished Phase I homes. Phase II infrastructure construction begins.
**2008**	Families begin moving into rental and for-sale housing in Phase II section of the neighborhood.
**2009**	Natural drainage construction completed and High Point reconnected to West Seattle services.
**2010**	Construction of all rental housing is completed. Private home-ownership development continues until the nearly 1,700-unit capacity of the site is reached.

a Source: Seattle Housing Authority (21).

b http://www.seattlehousing.org/redevelopment/high-point/plan/.

Families began moving into rental and for-sale housing in Phase II of the neighborhood redevelopment, which began in 2006 and was completed in 2010. Seven hundred and sixteen units were ultimately replaced. Three hundred fifty of these are operated by Seattle Housing Authority and serve extremely low-income households. Private home development will continue until the nearly 1,700-unit capacity of the site is reached. In addition, Providence Health Systems built and leased Elizabeth House, a 75-unit supportive housing program for low-income elderly residents. The community engagement process created plans for a new, mixed-income community with health as its focus. High Point residents wanted the kind of healthy living conditions that wealthier neighborhoods usually take for granted.

### Stabilization

The renovation of High Point resulted in a green and sustainable community (http://www.seattlehousing.org/redevelopment/high-point/photos/). The neighborhood has energy-efficient housing, a community clinic, a public library, rebuilt community-raised garden beds, a community center that offers child care and employment placement services, an assisted living facility (Elizabeth House), lush green parks, and walkable sidewalks that enhance social interaction and physical activity. The redevelopment incorporated many sustainable development principles. The site and rental housing are certified at the highest Built Green levels. Nearly all rental housing and homeowner units are Energy Star (http://www.energystar.gov/) rated. The site features porous sidewalks and parking areas and the only porous pavement street in Washington State. The site is situated on 120 acres with an engineered natural drainage system, which uses the ground to filter rainwater instead channeling water into a traditional conveyance system. The rebuilt homes have ventilation systems designed to bring in fresh air from the outside. According to Takaro et al, “Even small particles that might be bad for health, such as diesel particulate or any pollen in the case of an asthmatic, will be filtered out so the air in the home is actually healthier than the air outside” (14).

**Figure 1:**
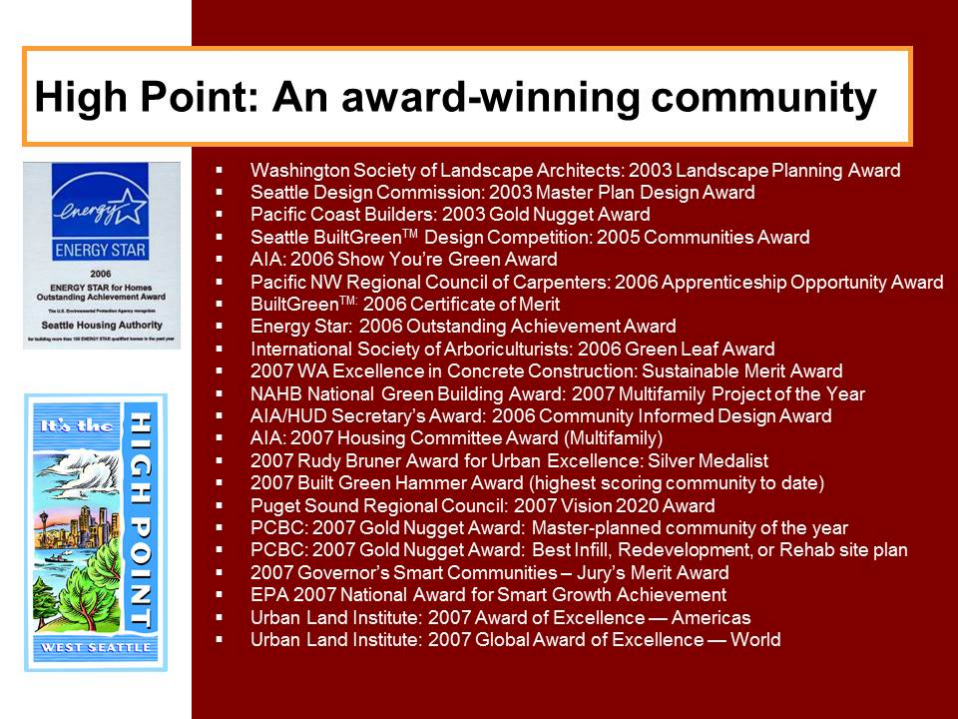
Illustration of the awards received by the High Point community. Abbreviations: AIA, American Institute of Architects; NAHB, National Association of Home Builders; HUD, US Department of Housing and Urban Development; PCBC, Pacific Coast Builders Conference; EPA, US Environmental Protection Agency.

The High Point neighborhood redevelopment has been recognized with prestigious land use and development awards (Figure 1) and has gained national attention for its progressive approach to redevelopment (10). In 2004, High Point was awarded the Pacific Coast Builders Conference’s prestigious Gold Nugget Award for “Best Plans on the Boards.” More recently, the American Institute of Architects honored the project with a coveted “Show You’re Green” award, 1 of only 8 in the nation awarded for sustainable innovations and affordability. High Point is also featured in 2 PBS documentaries, *Edens Lost and Found* and *Hidden Epidemics*. The former profiles redevelopment activities in Seattle, Los Angeles, Chicago, and Philadelphia, and the latter examines socioeconomic and racial disparities in health.

### Confirmation and expansion

By the end of the decade, High Point had nearly 1,700 new affordable and market-rate units. High Point includes housing units for residents with very low incomes (50% of area median income or below) and low incomes (80% of area median income or below) in addition to market-rate rental and for-sale housing. Most houses have private yards and porches. They sit on safe streets with controlled traffic and show great variety in architecture, character, and styles on each block. Proceeds from the land and home sales have helped fund low-income housing in the neighborhood and elsewhere. In addition, the land has been returned to the city’s property tax rolls, where it can generate revenues to help keep the neighborhood economically self-sufficient (10).

### Community ownership

High Point’s redevelopment success resulted from close cooperation among planners, residents, and other community stakeholders. All landscaping is well maintained, housing is uniform in style, and an observer would not detect differences between properties owned and properties rented. However, shrubbery is sometimes not properly trimmed; if it grows to sufficient heights, criminal activity may increase. Thus, continuous accountability and oversight are crucial, and all residents are responsible for making recommendations as needed relating to strategic planning, property management, and ongoing evaluation of customer services and needs to ensure the stability of the social and built environment.

## Outcome

### Improving air quality

Social science research has demonstrated a strong link between the social and built environments and their roles in contributing to healthy outcomes. For example, Takaro et al (14) examined the asthma-related clinical outcomes of living in High Point’s Breathe Easy Homes. A quasi-experimental design was used to compare the asthma outcomes of 2 groups of low-income children and adolescents with asthma. One group of 34 participants who moved into a Breathe Easy Home was matched with a second group of 68 local residents who had received a previous asthma-control intervention. Both groups received in-home asthma education. The group in the Breathe Easy Homes had less asthma-related illness than the comparison group, but most differences in improvements were not significant. The researchers reported that “Breathe Easy Home residents’ asthma-symptom–free days increased from a mean of 8.6 per 2 weeks in their old home to 12.4 after 1 year in the Breathe Easy Home.” Furthermore, the proportion of Breathe Easy Home residents with an urgent asthma-related clinical visit in the previous 3 months decreased from 62% to 21%. Breathe Easy Home caretakers’ quality of life improved significantly. Residents in the Breathe Easy Homes group improved more than did those in the comparison group, but most differences in improvements were not significant. However, exposures to mold, rodents, and moisture were reduced significantly. Children and adolescents with asthma who moved into an asthma-friendly home experienced large decreases in asthma morbidity and trigger exposure.

### Improving the built environment

As stated previously, High Point’s redevelopment plan was based on features of New Urbanism. These features, which contribute to a healthy community, include pedestrian friendly street designs (porches, hidden parking lots, wider sidewalks, tree-lined and slow-speed streets), a pond, multiple parks, walking trails, and separation of sidewalks from traffic by swales designed to manage storm water runoff and increase rainwater infiltration (14).

Using the Centers for Disease Control and Prevention’s (CDC’s) *Guide to Community Preventive Services* as a resource (19), community action teams developed multiple interventions to promote walking, including establishing walking groups, improving walking routes, providing information about walking options, and advocating for pedestrian safety. Self-reported walking increased among walking group participants from 65 to 108 minutes per day (13). The proportion of walking participants who reported being at least moderately active for at least 150 minutes per week increased from 62% to 81% in a 3-month period (13). General health improved, and walking group participants reported fewer days when physical health and mental health were not good.

### Improving the social environment

**Figure 2:**
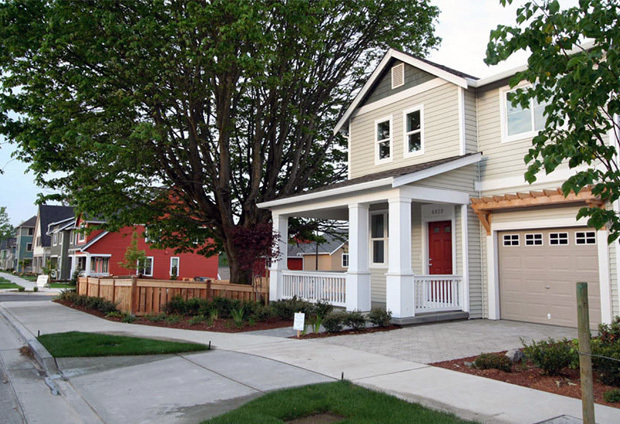
High Point’s narrow streets, short blocks, and wide planting strips promote walking. Front yards, porches located close to sidewalks, and the overall design of the community encourage social interaction. [A text version of this figure is available.]

The social environment affects physical activity through community safety, social support, and access to recreation and activity programs (13). Community action teams introduced the concept of walking groups as an effective social environmental strategy to promote physical activity. Teams modified the Walk Kit from the California Center for Physical Activity (http://www.caactivecommunities.org/resources/walk-kit/). High Point’s walkers were encouraged to meet *Healthy People 2010* recommendations of at least 30 minutes of moderate physical activity **(**ie, 30 minutes of exercise or 10,000 walking steps) on 5 or more days per week (13). The walking groups met 5 times per week at weekday, evening, and weekend sessions. Walking appears to have collateral benefits. Participants reported walking more for errands, and social interactions with their neighbors increased. Walkers received T-shirts, pedometers, and prizes as incentives for meeting individual walking goals. The walking sessions promoted walking among other community members who saw their neighbors walking together in a group identified by the T-shirts or bright yellow rain ponchos they were wearing (Figure 2).

## Interpretation

Housing matters for health (1–8), and quality housing is associated with positive physical and mental well-being (20). There is broad consensus that residents of socially and economically disadvantaged communities experience, on average, worse health outcomes than those living in more prosperous areas. The HOPE VI Urban Revitalization Demonstration Program’s housing initiative, with its explicit goal of decentralizing poverty, has demonstrated the benefits of reducing neighborhood-level poverty by providing affordable housing to low-income families in mixed-income residential developments. Relocation of residents may reduce the poverty level of an entire neighborhood but not necessarily the poverty level of individuals and particular families. As we move forward, more focus is needed on supporting collaborative programs that increase health equity across a range of health outcomes.

Efforts to create social change and improve health outcomes for entire populations have greater impact when many organizations work together to promote health causes. Consensus and collaboration among the affected population, with attention to its cultures, beliefs, and resources, should be included in planning strategies to improve and sustain living conditions related to resident health and public health issues. When the community is engaged, planning efforts are more likely to be based on concepts and ideas that are culturally appropriate for that unique community. To be successful, interventions should be tailored to the stage of readiness of the affected population to tackle complex public health issues. This case study of the High Point redevelopment project illustrates how the involvement and commitment of local residents in the planning and implementation of a local housing improvement effort can contribute to its success.

## References

[1] Hood E . How poor housing leads to poor health. Environ Health Perspect 2005;113(5):A310–7. 10.1289/ehp.113-a310 15866753 PMC1257572

[2] Acevedo-Garcia D , Osypuk TL , Werbel RE , Meara ER , Cutler DM , Berkman LF . Does housing mobility policy improve health? http://www.nchh.org/Portals/0/Contents/Article0668.pdf. Accessed September 14, 2014.

[3] Kramer MR , Hogue CR . Is segregation bad for your health? Epidemiol Rev 2009;31(1):178–94. 10.1093/epirev/mxp001 19465747 PMC4362512

[4] Osypuk TL , Acevedo-Garcia D . Beyond individual neighborhoods: a geography of opportunity perspective for understanding racial/ethnic health disparities. Health Place 2010;16(6):1113–23. 10.1016/j.healthplace.2010.07.002 20705500 PMC2952656

[5] Dunn JR . Housing and health inequalities: review and prospects for research. Housing Stud 2000;15(3):341–66. 10.1080/02673030050009221

[6] Acevedo-Garcia D , Lochner KA , Osypuk TL , Subramanian SV . Future directions in residential segregation and health research: a multilevel approach. Am J Public Health 2003;93(2):215–21. 10.2105/AJPH.93.2.215 12554572 PMC1447719

[7] Jacobs DE , Brown MJ , Baeder A , Sucosky MS , Margolis S , Hershovitz J , A systematic review of housing interventions and health: introduction, methods, and summary findings. J Public Health Manag Pract 2010;16(5):S5–10. 10.1097/PHH.0b013e3181e31d09 20689375

[8] US Department of Health and Human Services. The Surgeon General’s call to action to promote healthy homes. Washington (DC): Office of the Surgeon General; 2009. http://www.ncbi.nlm.nih.gov/books/NBK44192/pdf/TOC.pdf. Accessed August 1, 2014. 20669408

[9] Commission on Social Determinants of Health. Closing the gap in a generation: health equity through action on the social determinants of health: final report of the Commission on Social Determinants of Health. Geneva (CH): World Health Organization; 2008. http://www.who.int/social_determinants/thecommission/finalreport/en/. Accessed June 18, 2013.

[10] Kleit RG , Allison J . HOPE VI for High Point: baseline report. Seattle (WA): Daniel J. Evans School of Public Affairs, University of Washington. https://www.ksamedia.osu.edu/sites/default/files/originals/Kleit-9.pdf .

[11] Edwards RW , Jumper-Thurman P , Plested BA , Oetting ER , Swanson L . Community readiness: research to practice. J Community Psychol 2000;28(3):291–307. 10.1002/(SICI)1520-6629(200005)28:3<291::AID-JCOP5>3.0.CO;2-9

[12] Plested BA , Edwards RW , Jumper-Thurman P . Community readiness: a handbook for successful change; 2006. Fort Collins (CO): Tri-Ethnic Center for Prevention Research.

[R13] Krieger J , Rabkin J , Sharify D , Song L . High Point walking for health: creating built and social environments to support walking in a public housing community. Am J Public Health 2009;99(Suppl 3):S593–9. 10.2105/AJPH.2009.164384 19890163 PMC2774172

[14] Takaro TK , Krieger J , Song L , Sharify D , Beaudet N . The Breathe-Easy Home: the impact of asthma-friendly home construction on clinical outcomes and trigger exposure. Am J Public Health 2011;101(1):55–62. 10.2105/AJPH.2010.300008 21148715 PMC3000722

[15] Israel BA , Schulz AJ , Parker EA , Becker AB . Community-based participatory research: policy recommendations for promoting a partnership approach in health research. Educ Health (Abingdon) 2001;14(2):182–97. 10.1080/13576280110051055 14742017

[16] Carter JS . Reassessing the effect of urbanism and regionalism: a comparison of different indicators of racial tolerance. Sociation Today 2005; 3(3). http://www.ncsociology.org/sociationtoday/v32/urbanism.htm. Accessed September 23, 2014.

[17] Mumford KG , Contant CK , Weissman J , Wolf J , Glanz K . Changes in physical activity and travel behaviors in residents of a mixed-use development. Am J Prev Med 2011;41(5):504–7. 10.1016/j.amepre.2011.07.016 22011422

[18] Farr Associates. An expert review on the strength of the public health data in support of proposed community design standards in LEED for neighborhood development; 2008. http://www.farrside.com/media/CDC-LEED-ND_Report2.pdf. Accessed August 1, 2014.

[19] Centers for Disease Control and Prevention. Guide to community preventive services. Atlanta (GA): US Department of Health and Human Services; 2014. http://www.thecommunityguide.org/pa/index.html. Accessed November 18, 2013.

[20] National Prevention Council. National prevention strategy. Washington (DC): US Department of Health and Human Services, Office of the Surgeon General; 2011. http://www.surgeongeneral.gov/initiatives/prevention/strategy/report.pdf. Accessed August 1, 2014.

[21] Seattle Housing Authority. Redevelopment plan. http://www.seattlehousing.org/redevelopment/high-point/plan/. Accessed September 14, 2014.

